# Trends in Primary Anatomical Single-Bundle Anterior Cruciate Ligament Reconstruction Practice in Adult Patients Prevalent Among Arthroscopy Surgeons of Six Southern States of India

**DOI:** 10.1007/s43465-022-00719-z

**Published:** 2022-08-20

**Authors:** Vivek Pandey, Sandesh Madi, Chirag Thonse, Clement Joseph, David Rajan, Jacob Varughese, Jai Thilak, P. S. Jayaprasad, Kiran Acharya, Krishna Gopal Ramamurthy, Raghuveer Reddy, Rajkumar Amravathi, Sharath Rao, Sridhar Gangavarapu, Moparthi Srinivas, Sujit Jose, S. R. Sundararjan

**Affiliations:** 1 Department of Orthopaedics, Kasturba Medical College, Manipal, Manipal Academy of Higher Education, Manipal, 576104 India; 2 Vikram Hospital, Millers Road, Bangalore, 560052 India; 3 Arthroscopy and Sports Medicine, SRM Institute for Medical Sciences, Vadapalani, Chennai, Tamil Nadu 600 026 India; 4 Ortho-One Orthopaedic Speciality Centre, Trichy Road, Singanallur, Coimbatore, Tamil Nadu 641005 India; 5 VPS Lakeshore Hospital, Kochi, Kerala India; 6 Amrita Institute of Medical Sciences and Research Centre, Kochi, Kerala 682 041 India; 7 Kamineni Hospitals, L.B. Nagar, Bahadurguda, Hyderabad, Telangana 500068 India; 8 Brindavanam, Puducherry, Puducherry, 605013 India; 9 Krishna Institute of Medical Sciences Secunderabad, Hyderabad, India; 10 Care Hospitals in Banjara Hills, Hyderabad, India; 11 Sai Institute of Sports Injury and Arthroscopy in Banjara Hills, Hyderabad, 500 004 India; 12 St John’s Medical College Hospital, Sarjapur Road, Bangalore, 560034 India; 13 Department of Orthopaedics, Medicover Hospitals, Gokhale Rd, Krishna Nagar, Maharani Peta, Visakhapatnam, Andhra Pradesh 530002 India; 14 Nandan Hospital, Suryarao Pet, Vijayawada, Andhra Pradesh 520002 India; 15 Institute of Advanced Orthopaedics at MOSC Hospital and Medical College, Kolenchery, Ernakulam, Kerala 682311 India; 16 Arthroscopy and Sports Medicine, Ganga Hospital, Coimbatore, 641 043 India

**Keywords:** Anterior cruciate ligament reconstruction, Single bundle, Arthroscopy, Survey, Trend

## Abstract

**Background:**

Although guidelines from multiple scientific studies decide the general trend in ACLR practice, there is often a variation between scientific guidelines and actual practice.

**Methods:**

A 17-member committee comprised of sports surgeons with experience of a minimum of 10 years of arthroscopy surgery finalized a survey questionnaire consisting of concepts in ACL tear management and perioperative trends, intraoperative and post-operative practices regarding single-bundle anatomic ACLR. The survey questionnaire was mailed to 584 registered sports surgeons in six states of south India. A single, non-modifiable response was collected from each member and analyzed.

**Results:**

324 responses were received out of 584 members. A strong consensus was present regarding Hamstring tendons preference for ACLR, graft diameter ≥ 7.5 mm, viewing femoral footprint through the anterolateral portal, drilling femoral tunnel from anteromedial portal guided by ridges and remnants of femoral footprint using a freehand technique, suspensory devices to fix the graft in femur and interference screw in the tibia and post-operative bracing. A broad consensus was achieved in using a brace to minimize symptoms of instability of an ACL tear and antibiotic soaking of graft. There was no consensus regarding the timing of ACLR, preferred graft in athletes, pre-tensioning, extra-articular procedure, and return to sports. There was disagreement over hybrid tibial fixation and suture tapes to augment graft.

**Conclusion:**

Diverse practices continue to prevail in the management of ACL injuries. However, some of the consensuses reached in this survey match global practices. Contrasting or inconclusive practices should be explored for potential future research.

## Introduction

Currently, ACL tears constitute a significant proportion of knee ligament injuries. Unlike the US or European countries, where a national registry is maintained to document the exact incidence of these injuries, in countries like India, sparse epidemiological data exists. In 2014, John et al. noted that ACL tears constituted 86.5% of all knee injuries among sportsperson from Northern India [1]. However, the overall incidence of ACL tears in the general population and other parts of the country is vastly unknown. Furthermore, there is also a paucity of information on the trends and practices in ACL management from this part of the world.

The outcome of ACL reconstruction (ACLR) depends upon various preoperative, intraoperative, and post-operative factors, including rehabilitation. Globally, surgeons refer to published literature from various sources to decide upon decision-making regarding the timing and type of surgery, type, and diameter of graft, surgical technique, fixation devices, post-operative bracing, and return to sport to optimize the outcome. Despite the presence of published evidence, many ‘other practical factors’ often influence decision-making, such as fellowship training, regional or institutional practices, the impact of conferences and workshops, and unpublished anecdotal personal experiences of the surgeon. Furthermore, the enormous number of scientific studies published worldwide and the continuously evolving concepts around the management of ACL tears have led to diverse practices and surgical protocols being followed for the same type of injury. Recently, an international, multidisciplinary group of experts (Panther Symposium ACL Treatment Consensus Group) also noted that the evidence supporting best-practice guidelines for managing ACL injury is primarily based on low-level evidence studies [2].

A well-structured survey performed among active practitioners would provide a great insight into the rationale for these practices and whether the practitioners follow the global trends or vary from their peers. Further, this survey will serve as a platform for potential future studies to address the practice gap and develop strong uniform adoption guidelines.

Many such surveys have been conducted worldwide to understand the nuances in ACLR practice (Table 1). There seems to be consensus on many fronts, while variation exists on several others regarding ACLR. However, no such surveys have been conducted in India to understand the diversity of this commonly performed procedure among Arthroscopic surgeons. In most surveys, several aspects of the ACLR practice trend have not been explored, such as pre-soaking the graft with antibiotic, pre-tensioning, footprint identification, post-operative bracing, etc. (Table 1). The purpose of this questionnaire-based online survey among the arthroscopic surgeons of six south Indian states was to determine the current trends and practices in common issues regarding primary single-bundle anatomical ACLR and compare it with worldwide prevalent trends and practices.

**Table 1:** Parameter assessed in various surveys worldwide

Author, ref (year)	Graft choice athlete	Graft choice non-athlete	Antibiotic pre-soaking	Surgical technique	Portal for femoral tunnel placement	Femoral footprint identification	Graft pre-tensioning	Femoral fixation	Tibial fixation	Knee position while fixing graft	Postop DVT prophylaxis	Postop immediate weight-bearing	Postop bracing	RTS timing (6–9 months)	DOI^a^
McRae et al. [39] (2011)	NA	HT- 73%	NA	54% SB	70%- TT	NA	82%- M	51%- SF	63.2% BAS	30^0^- 39.7%	NA	72.10%	48.5%	56.30%	
Chechik et al. (2011)	NA	HT- 63%	NA	67% SB	68% AMP	NA	NA	40%- SF	NA	NA	NA	NA	NA	NA	10.1007/s00264-012-1611-9
Mahnik et al. [34] (2013)	NA	HT- 95%	NA	NA	67%-AAM	NA	NA	62%- SF	97% BAS	NA	NA	25.64%	66.67%	66.67%	
Farber et al. [37] (2014)	BPTB-68%	NA	NA	91% SB	50%-TT	NA	NA	NA	NA	NA	NA	NA	32%	82.00%	
Erickson et al. [10] (2014)	45.3–86.1% BTB	NA	NA	99.3% SB	67%- AAM	NA	NA	NA	NA	NA	NA	NA	35.77%	55.47%	
Ambra et al. (2015)	NA	HT- 93%	NA	NA	50%- AAM	NA	NA	NA	NA	NA	NA	NA	NA	NA	10.1007/s00264-015-2905-5
Kirwan et al. (2015)	NA	HT- 92.4%	NA	NA	NA	NA	80%- M	NA	NA	NA	NA	NA	NA	NA	10.1007/s00402-015-2335-2
Van der Bracht et al. [36] (2015)	NA	HT- 91%	NA	93% only SB	58%-AM	NA	NA	91%- SF	91% Screw; Hybrid fixation- 64.4%	NA	NA	53.30%	70.7%	NA	
Grassi et al. (2016)	49% HT	HT- 81%	NA	NA	62%- TT	NA	NA	NA	NA	NA	NA	NA	NA	NCS: 92%; CS- 72%	10.1055/s-0038-1672157
Budny et al. [33] (2016)	61% of male athletes: BPTB	HT- 45%	NA	92.3% SB	47%-AM	NA	NA	79%- SF in HT; 79.4% Screws (BPTB)	85.9% Screws (HS): 98.1% Screws (BPTB)	NA	47.7% Yes	68.70%	85.3	65.5-BPTB; 63.6%- HT	
Vaishya et al. [35] (2016)	NA	HT-83.3%	NA	83.3% SB	86.9%- AMP	NA	NA	93.75%- SF	95.83% Screws	NA	NA	68.6% (EWB)	85.4%	NA	
Lynch et al. (2020)	NA	BPTB-51.5%	NA	NS	OI technique 76.3%	NA	NA	62.4%- SF	78.9% screw	NA	NA	NA	NA	NA	10.1016/j.asmr.2020.06.003
Koc et al. (2020)	NA	HT-87.2%	NA	NA	50.4%- AMP	NA	NA	NA	NA	NA	NA	NA	NA	75.20%	10.1016/j.jcot.2020.02.002
Arnold et al. (2021)	NA	HT > 50%'	NA	NA	NA	NA	NA	NA	NA	NA	NA	NA	NA	NA	10.1007/s00167-021-06443-9

*NA* not available, *NS* not specified, *HT* hamstring tendon, *BPTB* bone–patellar tendon–bone, *SB* single bundle, *TT* transtibial, *AMP* anteromedial portal, *AAMP* accessory anteromedial portal, *OI* outside-in, *M* manual, *SF* suspensory fixation, *BAS* bioabsorbable screws, *NCS* non-contact sports, *CS* contact sports

^a^DOI is indicated for the references which are not included in the main list of references in the manuscript

## Materials and Methods

After the initiative taken on behalf of the scientific committee of the Orthopaedic Association of six states of south India, the primary research group selected 2–3 coordinators per state who had more than ten years of experience in Arthroscopy regarding ACLR. The entire academic group comprised 17 surgeons who further developed a preliminary 30 questions based upon various issues of ACL management. These questions covered relevant preoperative, intraoperative, post-operative, and return to sports issues in ACLR. After considerable deliberations, discussions, and suggestions, a questionnaire was decided. The content validation of questionnaire (content determination, item generation, instrument construction, judgement, and determination of content validity index) was done with seven sports injury arthroscopy surgeons who had an experience of more than ten years in the field of managing ACL injuries [3]. Once the questionnaire was validated, it was entered into an electronic form based upon google form (Google LLC, California, USA) (Table 2).

**Table 2:** Survey questionnaire

Section 1 1. Experience in arthroscopy in years; 2. Workplace; 3. The average number of primary ACL reconstructions performed per year Section 2: Beliefs in ACL tear management and Perioperative trends in ACL practice 1. During conservative treatment, bracing is useful to minimize symptoms of ACL insufficiency. 2. ACL tear, if untreated for long, is associated with knee arthrosis. 3. ACL reconstruction reduces the rate of arthrosis compared to non-reconstructed cases. 4. Do you also perform double-bundle ACL reconstruction and Primary ACL repairs? 5. Have you been performing any newer technique of ACL reconstruction such as 'All-inside' ACL reconstruction? 6. Do you believe in ACL reconstruction in the acute phase (< 3 weeks) Section 3: Intra-operative trends 1. What is your most preferred graft in a non-athlete? 2. What is your most preferred graft in an athlete /other high-demand patients such as commandos etc.? 3. What is the minimum diameter of the ACL graft (especially Hamstrings) you accept for reconstruction? 4. How do you increase the diameter of the Hamstring graft especially if quadrupled semitendinosus is less than 7.5/8 mm? 5. If the soft tissue graft diameter is not acceptable, do you ever augment the graft with a 'fibre tape' like the material? 6. Do you perform 10–15 N pre-tensioning of soft ACL graft? 7. Do you soak the graft in antibiotic for 10–15 min before pulling it up in the knee? 8. If the answer to the above question is yes, then which antibiotic? (Otherwise, skip the question). 9. Which portal do you use to visualize and further identify the femoral footprint of ACL? 10. Which portal do you use to establish a femoral tunnel? 11. Which technique do you use to identify the center of femoral footprint? 12. Do you measure the tibial footprint? 13. Do you believe in saving the residual tibial stump as much as possible to enhance so-called proprioception? 14. Which is your most preferred femoral fixation method for the graft (soft tissue and Bony plug ones)? 15. What is your most preferred tibial fixation method? 16. Do you 'often' use additional fixation in soft grafts for the tibial side such as staple/screw post, etc.? 17. When do you add extra-articular procedures in primary ACL reconstruction such as ALL reconstruction/IT band strip tenodesis (modified Lemaire procedure)? Section 4: Post-operative trends 1. Do you use brace do you prefer to use in the post-operative phase, and type of brace? 2. In no meniscal or cartilage repair scenario, what is your weight-bearing protocol in such cases? 3. How do you decide to return to sports in the athlete/high-demand patients? 4. When do you decide to return to sports?	

*ACL* anterior cruciate ligament, *ALL* anterolateral ligament, *IT* iliotibial

The idea of the questionnaire was to determine the trend of each practice in single-bundle ACLR and assess possible consensus on each of those parameters. We defined consensus as the following: strong consensus if more than 75% of responders agreed to a particular practice, broad consensus at 60–74.9% of responses, inconclusive between 40 and 59.9%, and disagreement if less than 40% of responders agreed to a particular practice [4].

Once the survey questionnaire was finalized, state coordinators circulated the google form to the registered arthroscopy surgeons of their state via e-mail and WhatsApp. While consenting to participate in the survey, the participants were informed that the survey was being conducted as a part of OASIS scientific committee initiative to understand nuances of variations in the practice of ACL surgery and trends in ACL management. The participants were informed that the form carries 30 questions spread over five pages (sections), and it would take approximately 10 min to complete the form. All questions were compulsory, and, therefore, the form could not be submitted till all questions were answered. The participants were also informed that the data would be kept confidential and deleted once the analysis followed by publication was completed. The form could be filled by a member once only and was not editable once submitted. However, a copy of the submitted response was emailed back to the member. No cookies or IP address identification systems were used to identify the user (member). The survey was continued for ten days while reminder to registered members was sent every 3rd day. The data obtained were stored in a Microsoft Excel sheet, which was accessible only to the principal investigator of the survey for the purpose of data protection.

## Results

**Fig. 1:**
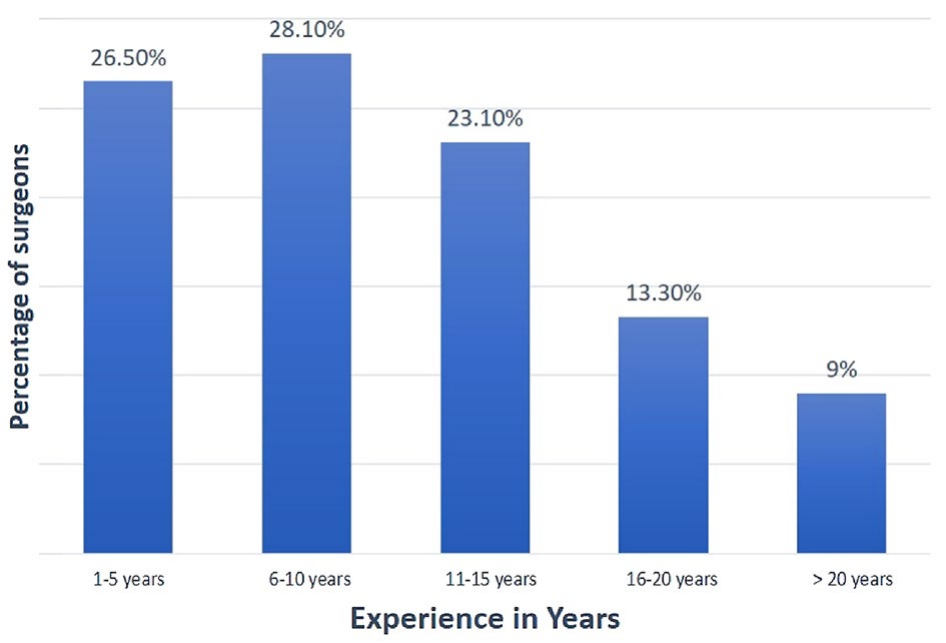
Bar chart showing experience (in years) of performing arthroscopic ACL reconstruction of surgeons who participated in the survey

**Fig. 2:**
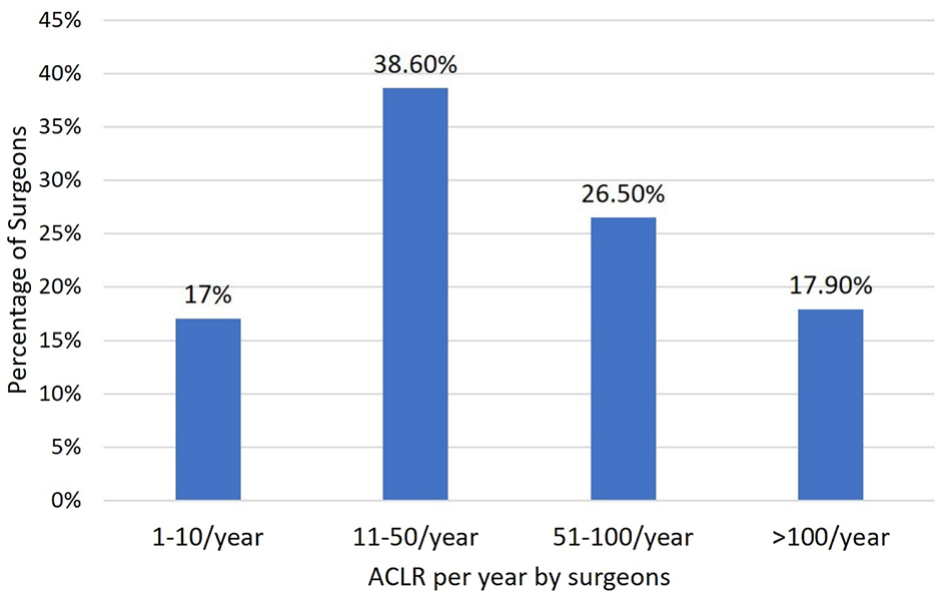
Bar chart showing number of ACL reconstructions performed per year by the surgeons who participated in the survey

After completing the survey, we received 324 responses out of 580 registered arthroscopy surgeons. Survey results were analyzed and summarized into tables, and a consensus was assessed based on the above criteria. 45.4% of surgeons who participated in the survey had an experience of more than 10 years (Fig. 1). Many surgeons (38.6%) performed 11–50 ACLR per annum, followed by 26.5% of surgeons who performed 50–100 cases per annum (Fig. 2).

### Preoperative Trends (Table 3)

**Table 3:** Results of beliefs in managing ACL tear and perioperative practices in managing ACL tear

	Beliefs in managing ACL tear and Perioperative decision-making regarding ACLR	Preferences			
1	Unmanaged ACL tear results in OA knee in long run	Yes—85.8%		No—14.2%	
2	ACLR prevents OA knee compared to non- reconstructed ACL tear	Yes—77.5%		No—22.5%	
3	Bracing minimizes the instability	Yes—69.1%		No—30.9%	
1	Performing ACLR in acute phase (< 3 weeks)	Yes—51.5%		No—48.5%	
2	Techniques of performing ACLR other than standard SB ACLR	*DB ACLR*	Never—76.8%	*All—inside* *ACLR*	Never—52.8%
			Occasional—16.9%		Occasional—33.6%
			Frequent—6.17%		Frequent—13.6%
3	Ever performed ACL repairs	Never—44.13%; Occasional—31.8%; Frequent—24.1%			

*ACLR* anterior cruciate ligament reconstruction, *DB* double bundle, *SB* single bundle, *OA* osteoarthritis

85.8% of surgeons believed that untreated ACL tear results in knee osteoarthritis, and 77.5% believed that ACLR prevents the development of OA. Nearly 70% of surgeons use a brace to minimize symptoms of instability in an ACL tear.

Further, 51.5% of surgeons prefer performing ACL reconstruction in the acute phase (< 3 weeks). Regarding techniques other than single-bundle ACLR, more than three-fourths of the surgeons surveyed had never performed double-bundle ACLR. 47.2% perform all-inside ACLR (13.6% frequently; 33.6% occasionally). Regarding ACL repair, 55.9% perform ACL repairs (24.1% frequently; 31.8% occasionally).

### Intra-operative Trends (Table 4)

**Table 4:** Results of intra-operative and post-operative practice trends

	Intra-operative trends	Preferences					
1	Most preferred graft in non-athletes	Hamstring—93.5%		Peroneus—3.4%		BPTB—2.5%	Quadriceps—0.6%
2	Most preferred graft in Athletes	Hamstring—51.2%		BPTB—41.7%		Peroneus—4.3%	Quadriceps—2.8%
3	Minimum graft diameter	8 mm–58.3%		7.5 mm–22.8%		7.0 mm–15.4%	
4	How to increase graft diameter?	Add Gracilis—85.8%		Accept the diameter as it is—8.6%			
5	Use of Fibretape like material for graft	Yes—38.9%		No—57.1%			
6	Performing Pre-tensioning of graft	Yes—54.9%		No (as surgeon does not believe in it)— 23.8%		Lack of tensioner− 21.3%	
7	Graft soakage in antibiotic	Yes—60%		No—40%			
8	Which antibiotic used for soaking graft	Gentamycin—64.1%		Vancomycin—32.3%			
9	Portal used to visualize femoral footprint	Standard AL portal—63.9%		Standard AL followed by AM portal—36.1%			
10	Technique used to identify femoral footprint	Free hand guided by ridges and footprint remnant—56.5%		Femoral offset method—34.3%		Malleable scale method—6.2%	Fluoroscopic method—1.2%
11	Portal used to drill femoral tunnel	Standard AM—63.3%		Accessory inferomedial—34.6%		Transtibial—2.2%	
12	Surgeons measuring tibial footprint	Yes—79%		No—21%			
13	Believe in Saving residual tibial footprint	Yes—83.6%		No—16.4%			
14	Most preferred femoral fixation method	*Soft* *Graft*	Suspensory variable loop—47.83% Suspensory fixed loop—39.81% Interference screw—10.18%	*Bony* *Graft*	Interference screw—87.34% Suspensory variable loop—6.17% Suspensory fixed loop—5.24%		
15	Most preferred Tibial fixation method Irrespective of type of graft	Interference screw—93.2%		Suture disc—6.8%			
16	Hybrid fixation in tibia	Yes—20.1%		No—79.9%			
17	Additional EAP	Yes—52.8%		No—45.4%			

	Post-operative trends	Preferences			
1	Use of knee brace (KI/HB/ACLB)	Yes—89.2% (KI:HB = 54%:28.4%:6.8%)	No brace—10.8%		
2	Weight bearing in absence of meniscal/cartilage lesion	Full weight-bearing in brace with EC—48.8%	Partial weight-bearing with AC—43.3%	NWB—7.9%	
3	Return to sports	After 9 months–46.3%	After 6 months—36.3%	> 12 month to 16.4%	
4	MRI to look for ACL healing before return to sports	No—96.6%			

*BPTB* Bone–patellar tendon–bone, *AL* anterolateral, *AM* anteromedial, *EAP* extra-articular procedure, *KI* knee immobilizer, *HB* hinge brace, *ACLB* ACL brace, *EC* elbow crutch, *AC* axillary crutch

- Graft diameter-related issues: 60% of surgeons in the survey preferred a minimum of 8 mm graft diameter, while the other 23% were comfortable if the minimum diameter was 7.5 mm. 57.1% of surgeons did not want to use artificial material like ‘fiber tape’ to enhance graft diameter.
- Regarding antibiotic usage to soak the graft: 58% of surgeons believed in soaking the graft in the antibiotic, and Gentamycin (64.1%) was preferred over Vancomycin (32.3%).
- Portals to visualize femoral footprint and establish femoral tunnel: 63.9% of surgeons preferred using only a standard anterolateral (AL) portal, while 36.1% initially used a standard AL portal followed by an anteromedial (AM) portal to visualize the femoral footprint. 56.5% of surgeons use the freehand technique guided by remnant femoral footprint and ridges (lateral intercondylar and bifurcate) to establish the femoral tunnel.
- Femoral–tibial fixation of graft and additional tibial fixation: 87.6% of surgeons preferred suspensory fixation over interference screw in the femoral tunnel for a soft graft. For bony grafts, 87.3% of surgeons prefer interference screw over suspensory devices. 93.2% of surgeons preferred interference screw for graft fixation in the tibial tunnel.
- Only 52.8% of surgeons prefer extra-articular procedures while preforming primary ACL reconstruction in patients who are into athletes in pivoting sports or with grade III pivot shift in absence of root tears (5.6%—athletes into pivoting or contact sports; 14.5%—grade III pivot shift test; and 32.7%—consider both options).

### *Post-operative Trends (Table **4**)*

Regarding decisions targetting return to sports (RTS): Most surgeons use a combination of clinical assessment, triple hop test, and the physiotherapist nod before allowing patients to return to sports. Most surgeons agreed not to ask for MRI to look for structural healing of ACL before RTS (96.6%). Regarding the timing of return to sports, 36.3% and 46.3% of surgeons keep six and nine months as cut-off time, respectively, while 16.4% of surgeons allow RTS after 12 months.

The consensus of all parameters assessed is mentioned in Table 5.

**Table 5:** Consensus statements among various parameters of Anterior cruciate ligament reconstruction

	Strong consensus (> 75%)	Broad consensus (60–74.9%)	Inconclusive (40–59.9%)	Disagreement (< 40%)
PREOPERATIVE	An unmanaged ACL tear may result in OA knee—85.8%	Bracing minimizes instability in ACL tear—69.1%	Performing ACL in acute phase vs. late 51.5% vs. 48.5%	
	ACLR may reduce the chance of OA knee—77.5%			
INTRAOPERATIVE	Hamstring tendon: Most preferred graft in non-athletes—93.5%	60% of surgeons use antibiotic to soak the graft	Hamstring vs. BPTB graft in athletes—51.2% vs. 41.7%	
		NO preference (57.1%) to use of synthetic suture material to enhance graft diameter (fiber tape)		
	Minimum graft diameter (≥ 7.5 mm)—81.1%	Gentamycin (64.1%) preferred over Vancomycin (32.3%)	Pre-tensioning of graft—54.9%	Preference to provide hybrid fixation on tibia—20.1%
	Add Gracilis to increase graft diameter—85.8%	Standard AL portal vs. AMP to visualize femoral footprint—63.9% vs. 36.1%	The technique used to identify the center of the femoral footprint	
	AM portal used to drill femoral tunnel—97.9%	Standard AM portal vs. AAM portal preferred to drill femoral tunnel: 63.3% vs. 34.6%	Additional EAP—52.8%	
	No surgical advantage in measuring tibial footprint for SB ACLR—79%			
	Saving residual tibial footprint is preferred—83.6%			
	Femoral tunnel fixation: Suspensory device is the most preferred method for soft graft—87.6%			
	Femoral tunnel fixation: Interference screw is the most preferred method for bony graft—87.3%			
	In tibial tunnel: Interference screw is the most preferred method for soft graft—93.2%			
POSTOPERATIVE	Preference to post-operative bracing—82.4%		FWB vs. PWB: 48.8% vs. 43.3%	
	Clinical and functional assessment along with therapist nod before RTS (94.4%)		Timing of Return to sports (after 6 months–46.3%; after 9 months–36.3%)	
	Do not ask for MRI to look for ACL healing before RTS (96.6%)			

*ACL* Anterior cruciate ligament, *OA* osteoarthritis, *AM* anteromedial, *AL* anterolateral, *SB* single bundle, *BPTB* bone–patellar tendon–bone, *EAP* extra-articular procedure, *FWB* full weight-bearing, *PWB* partial weight-bearing

## Discussion

This survey consolidates diverse opinions among arthroscopy surgeons of six southern states of India regarding pre-, intra-, and post-operative practices.

- *Beliefs in ACL tear management and perioperative decision-making*
  OA development after an ACL tear is multifactorial, and the evidence remains inconclusive in favor of operative versus conservative treatment [5]. However, the survey ended with a strong consensus that leaving ACL tear for long may result in osteoarthritis (OA) of the knee (85.8%), and ACLR would reduce the possibility of OA knee (77.5%).
  Our survey revealed that most surgeons (76.8%) never performed a double-bundle ACLR. The plausible reason behind the lack of performing DBACLR could be that most surgeons in our survey have only 10 years of experience in arthroscopy. Therefore, many were not exposed/trained to the DB ACLR technique as the trend of DB ACLR was declining in the last decade [6, 7]. Our survey also revealed the increasing trend of ACL repairs performed by surgeons. Once considered outdated, there is a renewed interest in ACL repair due to changes in techniques and a better biological understanding [8, 9].
- *Intra-operative trends*
  As per our survey, the Hamstring tendon (HT) autograft emerged as the most favored graft for primary ACLR in non-athletes with strong consensus, similar to other surveys (Table 1). However, the opinion in our survey remained inconclusive regarding the use of HT (51.2%) versus BPTB (41.7%) in athletes or other high-demand patients. A recent survey conducted among physicians of the United States associated with the National football league and major league soccer concluded that their preferred graft in athletes is BPTB (81–86%) [10]. Further, recent meta-analysis and systematic review indicate a slightly higher HT failure rate than BPTB [11]. Regarding the use of peroneus longus, our survey also showed that surgeons sparingly (4.3%) use the peroneus longus (PL) graft. A recent systematic review concluded that although the results of PL are comparable to HT, the possibility of a slight decrease in the American Orthopaedic Foot and Ankle Society scale score must be kept in mind while considering PL graft in primary ACLR [12].
  *Regarding graft diameter and reinforcing with sutures* Our survey revealed that the minimum graft diameter preferred by surgeons with strong consensus was 7.5 mm and above, while most preferred more than 8 mm (Table 4). Further, ‘assuming’ that surgeons were harvesting semitendinosus tondon (STT) first and quadrupling it, 85.8% of surgeons chose to add gracilis to the quadrupled STT to increase graft diameter if the ‘qaudrupled STT diameter was less than 7.5–8 mm. However, we did not collect data to find how many surgeons were directly taking STT and gracilis tendon together. Although a smaller diameter graft is at higher risk for rupture [11], the pertinent question is whether increasing graft diameter (> 8 mm) would result in a decreased rate of graft rupture or not, as several studies do not demonstrate any significant clinical benefit of increasing diameter above eight mm [13, 14]. Hence the clinical advantage of increasing the diameter of ‘soft graft’ beyond 8 mm should be further investigated with well-designed studies.
  Surgeons in our survey disagreed regarding the idea of reinforcing or augmentation of the graft with synthetic sutures. However, several clinical studies have suggested that graft augmentation with suture tape is associated with improved clinical outcomes and earlier return to preinjury activity level than standard HT ACLR without evidence of over constraint [15].
  *Regarding pre-tensioning of ACL* Our survey remained inconclusive regarding pre-tensioning of the graft. Although evidence regarding the clinical advantage of pre-tensioning remains elusive, biomechanical and laboratory studies demonstrate the potentially beneficial effect of pre-tensioning on the ACL graft, including reduced graft elongation, greater preservation of graft stiffness following fixation, and improved bone tunnel healing [16, 17].
  *Antibiotic usage for pre-soaking the ACL autograft* Our survey was broadly conclusive regarding pre-soaking the graft with antibiotic while preferring gentamycin over vancomycin. Recently, a survey of ACL international experts (116 surgeons) revealed that only 37.8% (25 out of 66 responders) used vancomycin to soak the graft [18]. The biggest concern for both surgeons who currently use and do not use vancomycin was the drug's effect on the mechanical properties of the graft, followed by cost and microbial resistance [18]. However, a recently published laboratory study suggests that there is no immediate adverse effect of vancomycin on the biomechanical properties of the graft [19].
  *Regarding the portal usage to visualize femoral footprint and establish femoral tunnel* Our survey has a broad consensus that surgeons prefer the AL portal over AM portal to visualize femoral footprint (Tables 4, 5). Although the AL portal remains a preferred portal to visualize and establish the femoral tunnel, several authors suggest visualization via AM portal as that provides an orthogonal view of the femoral footprint rather than an oblique or tangential view from the AL portal, and that may help in more precise identification of the footprint [20, 21]. However, a recently concluded study found no difference between the two techniques [22]. Nevertheless, well-designed studies are required to establish the superiority of AM over AL portal in ‘accurate’ identification of the femoral footprint as inaccurate tunnel placement remains one of the key factors in failed ACLR [23].
  *Regarding optimal technique in identifying the center of the femoral footprint* Our survey remained inconclusive regarding the preferred technique among surgeons to determine the center of the femoral footprint. In our survey, 56.5% surgeons used the freehand technique guided by ridges and residual stump, followed by offset (34.3%), malleable scale (6.2%), and rarely fluoroscopic method to identify the center of the femoral footprint. Present SB ACLR technique is based upon establishing the femoral tunnel in the center of the AM and PL bundles of the ACL [24]. Since femoral tunnel malposition is the most critical reason for ACL failures reported by 80% of surgeons [25], accurate femoral tunnel placement is one of the most vital steps in ACLR. Although the freehand technique guided by ridges and residual stump is most commonly used by our surgeons (56.5%), a laboratory study confirmed that the freehand technique, even in the hand of sports fellowship-trained surgeons, resulted in gross errors in femoral tunnel placement on a 3D model [26]. Furthermore, ridges (bifurcate and intercondylar) alone may not be the best option to identify the center of the femoral footprint because of variability in the visibility of ridges during arthroscopic surgery and anatomical variability of ridges [27].
  Regarding the use of offset, studies have shown a difference in femoral center location when assessed with standard over-the-top narrow offset versus broad offset [28]. With the variability in prevalent methods, combining two or more methods could be used to confirm the femoral tunnel location.
  *Regarding the use of portal while creating femoral tunnel* Recent studies indicate that creating an anatomic femoral tunnel is more precise using the AM portal than the transtibial [29], and that explains the shift in the surgeon’s approach to drilling the femoral tunnel through the medial portal compared to the other to the gold standard transtibial of the past. However, more clinical studies are required to confirm the superiority or similarity of the accessory AM portal over the standard AM portal in accurately creating the femoral tunnel.
  *Regarding the measurement of ACL tibial footprint* Most surgeons (80%) in our survey did not measure ACL tibial footprint. Perhaps, the reason for not measuring the tibial footprint could be that the ACL tibial footprint is relevant only when a surgeon has to decide between single- or double-bundle ACLR, as the latter can be performed if the tibial footprint is more than 14 mm [30]. Since most of our surgeons (76.8%) did not perform DB ACLR, the percentage of those measuring tibial footprint remained relatively low as that is not a must-to-do step in single-bundle ACLR.
  *Regarding tibial stump preservation* Strong consensus (83.6%) was observed in our survey regarding ACL stump preservation in anticipation that preserved stump will enhance proprioception and graft vascularisation. In contrast, other surveys have not assessed this practice (Table 1). A recent systematic review and meta-analyses have suggested that “remnant-preserving ACLR” promotes similar graft synovial coverage and revascularization to “standard ACLR” and comparable or superior clinical outcomes in the former [31].
  *Regarding femoral and tibial fixation of the graft* Our survey concluded a strong consensus for suspensory fixation (87.65%) for soft graft and aperture fixation or interference screw (87.34%) for a bony graft in the femoral tunnel. Other surveys, too, confirmed the same (Table 1). Although consensus between variable or fixed loop devices remained inconclusive, variable suspensory loop fixation was slightly preferred (47.83%) to fixed suspensory loop (39.81%).
  Irrespective of the type of graft, a strong consensus was observed regarding using an interference screw to fix the graft in the tibial tunnel.
  *Regarding hybrid fixation of graft on tibia* Surgeons in our survey strongly disagreed with providing hybrid tibial fixation (staple or screw post with washer over tibial cortex) other than interference screw in the tibial tunnel. Although several studies have concluded that hybrid fixation in the tibia results in stronger initial fixation and less side-to-side laxity, there is no clinical difference at 1- to 3-year follow-up [32]. The possible hesitation in providing hybrid fixation is that surgeons avoid another implant over the shin as it is ‘often proud’ or gives rise to a ‘remote chance of infection’ due to a proud implant just under the skin and adds to the additional cost and time.
  *Regarding extra-articular procedure (EAP)* The consensus remained inconclusive regarding adding an EAP (iliotibial band tenodesis or Anterolateral ligament reconstruction) even in specific indications such as high-grade pivot shift in the absence of meniscal root tear or patients who would return into pivoting sports or high-demand activities as only 52.8% of surgeons opted for EAP. Most recent surveys did not assess this aspect of ACLR practice (Table1). Recent reviews and meta-analysis indicate that combined primary ACLR and EAP are associated with improved outcomes when compared to isolated ACLR, including a significantly lower risk of ACL graft rupture and re-surgery for secondary meniscectomy following meniscal repair, improved functional outcome, and significantly increased likelihood of return to the preinjury level of the sport following primary ACLR [33].
- *Post-operative trends*

A strong consensus (89.2%) was observed in our survey regarding using one or another brace (Table 4) following ACLR in anticipation that the brace will provide additional support. Several surveys recommend bracing after ACLR [34–37], while others avoid using a post-operative brace [10, 38]. Even though post-operative braces appear to be popular among surgeons, recent systematic reviews and meta-analyses have suggest that routine knee bracing does not improve the clinical outcomes following ACLR [39].

*Regarding weight-bearing* after isolated ACLR, our surgeons remained inconclusive between full weight-bearing with a brace and partial weight-bearing for 3–4 weeks (Tables 4, 5). However, several surveys [34, 36, 40] and literature suggest full weight-bearing with support (crutch) and brace as long as there is minimal pain and no effusion with the correct gait pattern [41].

*Regarding return to sports (RTS),* a strong consensus among surgeons emerged, suggesting that the clinical and functional assessment of the patient along with the therapist nod is a must before the patient returns to sports. Recent reviews suggest a battery of clinical and psychological tests before the player is allowed to RTS [41]. However, considering the relevance of assessment of structural healing of ACL with MRI before RTS, only a minuscule (3.4%) of surgeons agreed on performing MRI alongside clinical evaluation before RTS.

*Regarding RTS timing,* surgeons remained inconclusive about whether the players should be allowed to RTS after 6 or 9 months or later. The RTS depends upon optimal clinical and functional restoration and a healed and matured ACL graft. Many recent studies have concluded that the hamstring ACL graft is not mature at six months, and RTS must be postponed at least nine months after the surgery to avoid graft re-rupture [42, 43].

*Limitation of the study* Although the survey covered close to 55% of registered surgeons who practice arthroscopy in six states, several other surgeons practice arthroscopy but are not registered with the society, along with surgeons who missed the survey resulting in a lesser response rate. However, a lower response rate in general surveys is a norm worldwide [18, 35]. Further, our survey's consensus in many ACL practices is similar to the worldwide pattern signifying that the sample covered is adequate. We did not attempt any non-response weighting or propensity scores to adjust non-responders of the survey. Second, the opinion of surgeons varies according to their experience level. However, a heterogeneous surgical population is a norm in all general surveys. Third, to cover broader aspects of ACL, not every detail could be asked, such as the type of screw preferred (metallic vs. bioabsorbable), the knee position while fixing the graft in the tibial tunnel, etc.

## Conclusions

For the first time, an Indian perspective on diverse aspects of practice and opinions on ACL management has been presented here. In sync with the evolving trends in ACL management, we have explored several updated features such as soaking the graft with antibiotic, pre-tensioning, footprint identification, using various portals, extra-articular procedures, and post-operative bracing conspicuously missing in previously conducted surveys in other parts of the world. While differences in practices and opinions will continue to exist, the results of this study provide a robust platform to explore the gaps and form a basis for future research.
